# Integrative analyses reveal transcriptome-proteome correlation in biological pathways and secondary metabolism clusters in *A. flavus* in response to temperature

**DOI:** 10.1038/srep14582

**Published:** 2015-09-29

**Authors:** Youhuang Bai, Sen Wang, Hong Zhong, Qi Yang, Feng Zhang, Zhenhong Zhuang, Jun Yuan, Xinyi Nie, Shihua Wang

**Affiliations:** 1 Key Laboratory of Pathogenic Fungi and Mycotoxins of Fujian Province, and School of Life Sciences, Fujian Agriculture and Forestry University, Fuzhou, 350002, China

## Abstract

To investigate the changes in transcript and relative protein levels in response to temperature, complementary transcriptomic and proteomic analyses were used to identify changes in *Aspergillus flavus* grown at 28 °C and 37 °C. A total of 3,886 proteins were identified, and 2,832 proteins were reliably quantified. A subset of 664 proteins was differentially expressed upon temperature changes and enriched in several Kyoto Encyclopedia of Genes and Genomes pathways: translation-related pathways, metabolic pathways, and biosynthesis of secondary metabolites. The changes in protein profiles showed low congruency with alterations in corresponding transcript levels, indicating that post-transcriptional processes play a critical role in regulating the protein level in *A. flavus*. The expression pattern of proteins and transcripts related to aflatoxin biosynthesis showed that most genes were up-regulated at both the protein and transcript level at 28 °C. Our data provide comprehensive quantitative proteome data of *A. flavus* at conducive and nonconducive temperatures.

*Aspergillus flavus* is a saprophytic filamentous fungus that is distributed all over the world especially in warm and moist fields^1^. It can produce an abundance of diverse secondary metabolites^2^, and the most well-studied group of metabolite is aflatoxin^3^, which includes AFB1, AFB2, AFG1 and AFG2^4^. Among these, AFB1 is predominant and the most carcinogenic and mutagenic polyketide; it contaminates a broad range of important agricultural crops including maize, wheat, peanuts, cottons, and nuts both before and after harvest^5^. These natural compounds not only affect grain growth and reproduction, but also cause significant economic losses of qualified yield in many countries.

Aflatoxin biosynthesis is a complex enzymatic reaction that has been extensively studied using available genome sequences of *A. flavus*^6^. Recently, a 70-kb gene cluster comprising 24 structural genes was identified involved in the biosynthetic pathway^7^,^8^. *A. flavus* is exposed to several environments, and aflatoxin production is controlled by several external factors and culture conditions, such as temperature, pH, water activity, and carbon and nitrogen source^9^. Recently our group reported the effect of water activity on the transcriptomic and proteomic profiles of *A. flavus* and dynamic changes of aflatoxin-related and development-related genes in different water activities^10^,^11^. Differential expression of noncoding RNAs (miRNA-like RNAs) was also identified in different temperature and water activities in *A. flavus*^12^. Furthermore, DNA methylation appears to be involved in aflatoxin metabolism and the development of *A. flavus*^13^.

One major determinant of aflatoxin production in *A. flavus* is temperature^14^,^15^; growth is favoured but aflatoxin production is not favoured at 37 °C, and the opposite is true at 28 °C^16^. Two fundamental strategies, termed “bottom-up” and “top-down” approaches, were used to identify proteins and quantify the changes at the proteome level of *A. flavus* in response to temperature changes^17^,^18^,^19^,^20^. However, how these changes are regulated is poorly understood^21^. The phenotypic aflatoxin contamination was clarified in several papers^22^,^23^, but very little information is available about the changes at the transcriptome–proteome level of *A. flavus* in response to temperature changes. The changes in the transcript and protein levels of *A. flavus* at 28 °C and 37 °C were profiled; the up-regulated proteins were enriched for translation-related pathways and the aflatoxin biosynthesis pathway. Our complementary transcriptome and proteome data indicate that post-transcriptional changes play a critical role in regulating the protein level in *A. flavus* in response to temperature changes.

## Results

### Transcriptome of *A. flavus* at 28 °C and 37 °C

**Figure 1:**
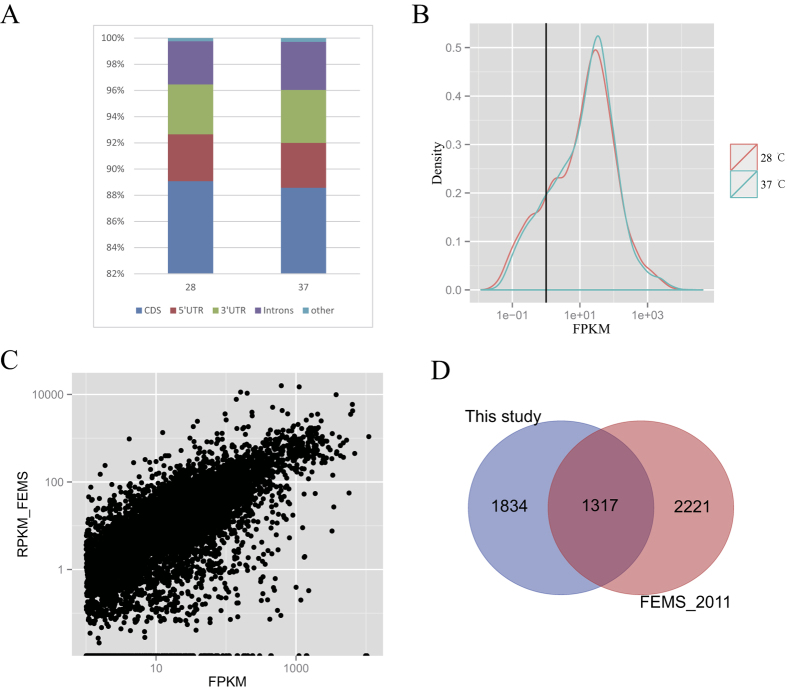
Transcriptome data of *A. flavus* at 28 °C and 37 °C. (**A**) RNA-Seq mapping statistics on different gene region. (**B**) Diagram of the FPKM value of genes. (**C**) Comparison of RNA seq data with Ref. 25 on 37 °C sample. (**D**) The number of DEGs identified by the two RNA seq dataset.

To study the effect of temperature on the transcriptome profile of *A. flavus, A. flavus* strain NRRL3357 was cultured at 28 °C and 37 °C and the isolated mRNA was subjected to high-throughput sequencing. In total, about 36 million 100-bp paired-end reads were obtained from the Illumina platform. Using the splice-aware aligner Tophat2^24^, 91.2% of reads were mapped to the *A. flavus* genome sequence, which represented much greater accuracy than earlier *A. flavus* transcriptome data^25^,^26^. More than 96% of reads were mapped to the exon region, including the 5′ untranslated region (UTR) and 3′ UTR region (Fig. 1A). This suggested that our RNA-seq data could precisely depict the transcription of protein coding genes in *A. flavus*.

The expression levels of transcripts were measured as fragments per kilobase of transcript per million mapped fragments (FPKM). The expression of *A. flavus* transcripts from both samples followed a bimodal distribution of high- and low-expression genes as described in other papers^27^,^28^, and 3,052 genes had an with FPKM value that was lower than 1 in both samples (Fig. 1B). After excluding these genes from further analysis, 75.8% of genes were detected expressed at 28 °C or 37 °C, which was similar to the results in previous studies in *A. flavus*^25^,^26^. Compared with the results of single-end RNA seq of *A. flavus* at 30 °C and 37 °C^25^, we found that the expression data of transcripts correlated well between samples (Spearman correlation coefficient, *rho* = 0.73 for samples grown at 37 °C samples [Fig. 1C] and *rho* = 0.75 for samples grown at 28 °C vs 30 °C). This indicated that our RNA-seq data comprehensively reflected the transcriptome profile of *A. flavus* at conducive and nonconducive temperatures.

To identify genes involved in temperature changes in *A. flavus*, differentially expressed genes (DEG) between the 28 °C and 37 °C samples in *A. flavus* were detected using the DEGseq tool. A total of 3,151 genes were significantly differentially transcribed between the 28 °C and 37 °C samples. We reanalysed the RNA seq data from 30 °C °C and 37 °C samples and identified 3,538 DEGs. Finally, 1,317 DEGs overlapped (Fig. 1D). Gene ontology (GO) annotation analysis showed that genes that highly responded to the temperature change in *A. flavus* were enriched in the following biological processes: “small molecule catabolic process”, “organic acid catabolic process”, “carboxylic acid catabolic process”, “cellular amino acid catabolic process”, “amine catabolic process” and “fatty acid metabolic process”.

### Annotation of proteome data

**Figure 2:**
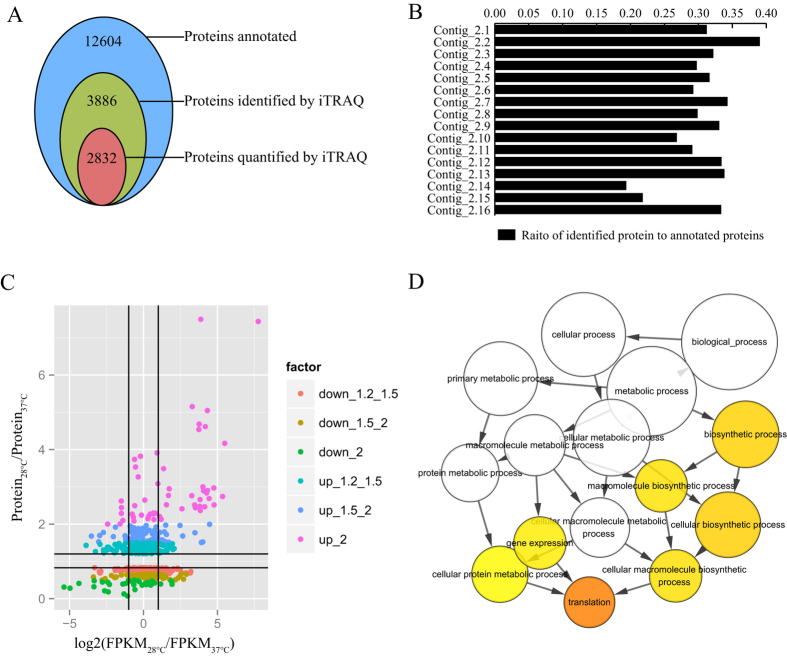
Annotation of proteome data. (**A**) The number of proteins identified and quantified by the iTRAQ method. (**B**) The number of protein identified were mapped into 16 contigs. (**C**) The differentially expressed proteins under temperature changes. (**D**) The GO term enrichment of the up-regulated proteins under 28 °C.

With regard to the proteomic response of *A. flavus* to temperature change, the proteome of *A. flavus* was quantitatively explored using the isobaric tags for relative and absolute quantitation (iTRAQ) technique. Proteins were extracted and digested in solution, then iTRAQ-labelled peptides were analysed by liquid chromatography combined with tandem mass spectroscopy (MS/MS). Total proteins in *A. flavus* were extracted from two biological experiments (28 °C and 37 °C) with three replicates. This experiment generated 270,924 spectra, of which, 33,406 spectra matched known peptides and 33,245 spectra matched unique peptides. Ultimately, 15,913 peptides, 15,862 unique peptides, and 3886 proteins were identified (Fig. 2A; Supplementary Table S1). We mapped 3880 of 3886 proteins to the 16 large contigs (Contig_2.1 to Contig_2.16), and we mapped three proteins to Contig_2.17 and one protein to each of Contig_2.27, Contig_2.29, and Contig_2.41. The proportion of identified proteins relative to all proteins for each contig varied, with the highest proportion of 39.03% on Contig_2.2 and the lowest proportion of 19.35% on Contig_2.14 (Fig. 2B). These results indicated that the genes that encoded functional proteins at 37 °C and 28 °C were not evenly distributed on 16 large contigs.

GO analysis showed that 2,864 proteins were annotated into different cellular components, including cell (25.2%), organelle (16.16%), macromolecular complex (9.85%) and membrane (7.47%). Processes such as “metabolic process” (35.37%) and “cellular process” (28.46%) made up considerable fractions of the total proteome, along with the important functional processes of localization, biological regulation, and response to stimulus. Such analyses revealed that a large fraction of the total protein was devoted to specific molecular functions including catalytic activity (48.81%) and binding (40.41%). About 60% of all identified proteins were assigned to 22 categories using the Clusters of Orthologous Groups (COG) database (Fig. 2C). The main functional categories were “General function prediction only” (27.62%), “Translation, ribosomal structure and biogenesis” (11.60%), “Post-translational modification, protein turnover and chaperones” (11.60%), “Amino acid transport and metabolism” (11.52%), “Energy production and conversion” (10.13%), “Carbohydrate transport and metabolism” (8.41%), “Transcription” (7.78%), and “Secondary metabolites biosynthesis, transport and catabolism” (7.31%).

Changes in the protein profiles in response to temperature changes in *A. flavus* were analysed. Replicate analyses revealed that our experiment using iTRAQ provided high accuracy in peptide quantitation. According to method suggested by Gan CS^29^, we found that more than 90% coverage of our identified proteins expression values fell within 50% expression variation (Supplementary Fig. S1). In total, 664 proteins from a total subset of 2,832 quantified proteins were identified as differentially expressed proteins (fold change >1.2 and P < 0.05; adjusted by false-discovery rate <5%). Among the proteins identified, 34, 126, and 327 proteins reproducibly produced less than 0.50-, 0.67-, and 0.83-fold at 28 °C compared to at 37 °C, respectively (Supplementary Table S2). Conversely, the levels of 55, 147, and 337 proteins increased by more than 2.0-, 1.5-, and 1.2-fold at 28 °C relative to 37 °C, respectively (Supplementary Table S2). The highest increase in abundance was observed for AFLA_034200 (2-heptaprenyl-1,4-naphthoquinone methyltransferase), AFLA_101580 (esterase/lipase), AFLA_064290 (predicted O-methyltransferase), AFLA_097310 (putative uncharacterized protein), AFLA_064450 (aminotransferase GliI-like, putative), and AFLA_139310 (aflE). Heat shock proteins (AFLA_037820 and AFLA_060260) and others showed the most pronounced down-regulation at 28 °C.

GO enrichment analysis revealed that up-regulated proteins (fold-change ≥1.2) at 28 °C were enriched in several biological processes including “translation” and “biosynthetic process”, while down-regulated proteins (fold-change ≤0.83) at 28 °C were not enriched in any GO term (Fig. 2D). Our analysis revealed high coverage (about 71% of the expressed proteins) within each KEGG pathway category. To identify differentially regulated biological processes between the two temperatures, we performed functional pathway enrichment analyses for differentially expressed proteins. The KEGG pathway “Ribosome” was strongly enriched among up-regulated proteins at 28 °C. These pathways included “Metabolic pathways”, “Carbohydrate metabolism”, and “Amino acid metabolism” (Table 1).

### Correlations between transcriptome and proteome data

**Figure 3:**
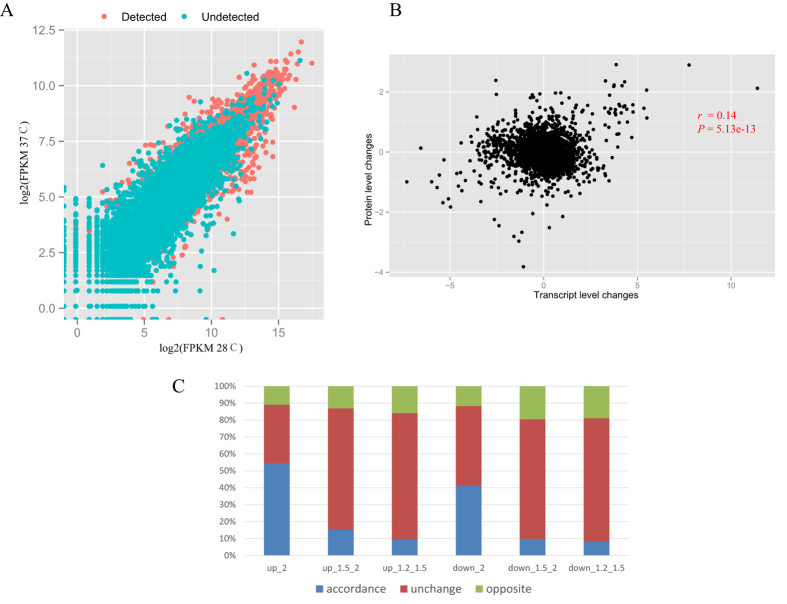
The correlation between the protein level and transcript level of genes in *A. flavus* at 28 °C and 37 °C. (**A**) Scatter diagram of the FPKM value of gene encoding the detected proteins. (**B**) Scatter diagram of the protein level changes and transcript level changes. (**C**) The proportional of the protein changes and transcript level changes.

It was reported that the mRNA abundance in the sample restricted identification of their cognate proteins^30^. At both 28 °C and 37 °C, the FPKM values of genes corresponding to identified proteins were significantly higher than those without detected proteins (Mann-Whitney-Wilcoxon Test, P < 2.2e-16; Fig. 3A). We observed increased coverage for proteins that were encoded by more abundant transcripts. Only two proteins that were detected by iTRAQ showed no read signals. Together, these data it is suggested that our proteomics data cover a very large part of the transcripts encoding functional proteins.

Next, we investigated whether changes in protein levels correlated with changes in the corresponding transcripts. A low correlation between transcript level changes and protein level changes was observed for all quantified proteins (Pearson correlation coefficient, *r* = 0.14, P = 5.13e-13, Fig. 3B) and differentially expressed proteins (*r* = 0.26, P = 5.26e-12). The differentially expressed proteins were divided into different fold changes: 1.2, 1.5, and 2. We found that an average of 16% of genes encoded a differentially expressed protein and showed direct conflict between the transcript level changes and protein level changes for the same gene. Additionally, we detected an increased proportion of proteins with large fold changes for both up- and down-regulated proteins (Fig. 3C). These results suggested that protein profiles that changed with different temperatures might be controlled at a post-transcriptional level, and changes in the mRNA expression provided only limited insight into changes in protein expression.

### Concordance within KEGG biological pathways

**Figure 4:**
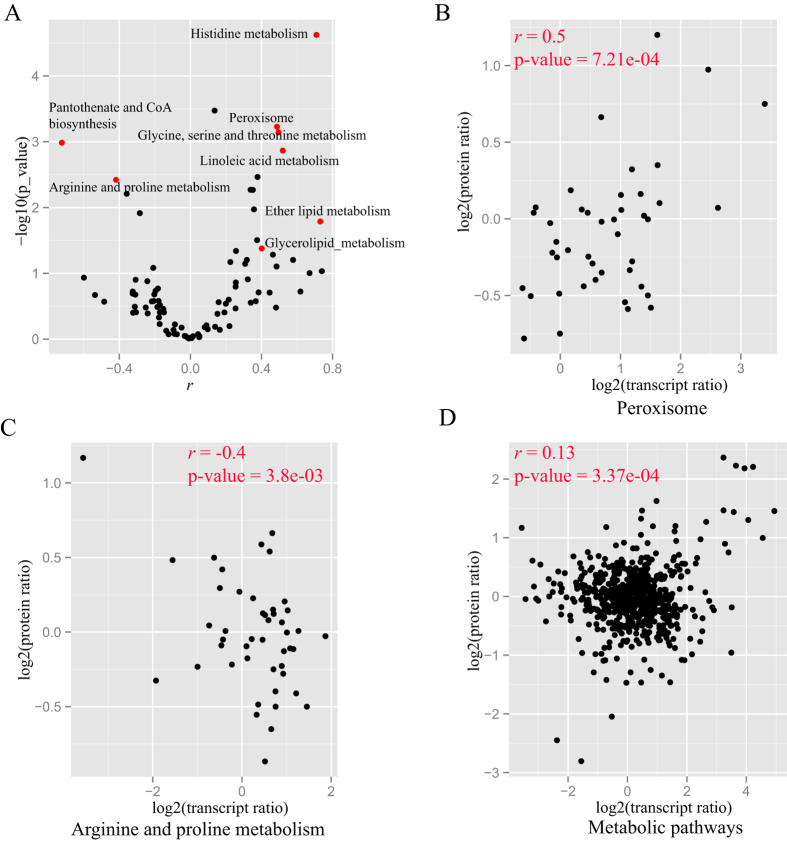
The correlation between the protein level and transcript level of genes within the KEGG pathway. (**A**) The overview of the correlation between the protein level and transcript level of genes within 92 KEGG pathways. Correlation between the protein level and transcript level of genes within “Peroxisome” (**B**), “Arginine and proline metabolism” (**C**) and “Metabolic pathways” (**D**).

We examined the level of concordance among transcripts and proteins of genes that are members of the same biological pathway. In total, 2,768 proteins were mapped to 108 biological pathways in KEGG. We performed correlation analyses on 94 pathways that contained more than five genes with both transcript and protein measured (Fig. 4A). We observed that some pathways, such as “Peroxisome” (Fig. 4B), had good concordance between changes of the transcript and protein levels (*r* > 0.4), indicating that changes in transcript expression cause corresponding changes in protein expression; hence, only minor alterations in the post-transcriptional regulation occur. Also, striking differences in the concordance between transcripts and proteins across some KEGG subcategories were observed. For example, for “Amino acid metabolism”, some KEGG subcategories (e.g. “Histidine metabolism” and “Glycine, serine and threonine metabolism”) generally showed a significantly strong correlation among the transcript and protein changes, while genes involved in “Arginine and proline metabolism” showed a significantly negative correlation (Fig. 4C; *r* = −0.4, P = 3.8e-03). The KEGG pathway “Metabolic pathways” generally showed significantly low correlations (*r* = 0.13, P = 3.37e-04; Fig. 4D). This indicated that proteins involved in metabolic pathways were particularly well controlled at the post-transcriptional level, and that changes in mRNA expression provided only limited insight into changes in protein expression.

### Expression patterns of the protein–protein interaction (PPI) network

**Figure 5:**
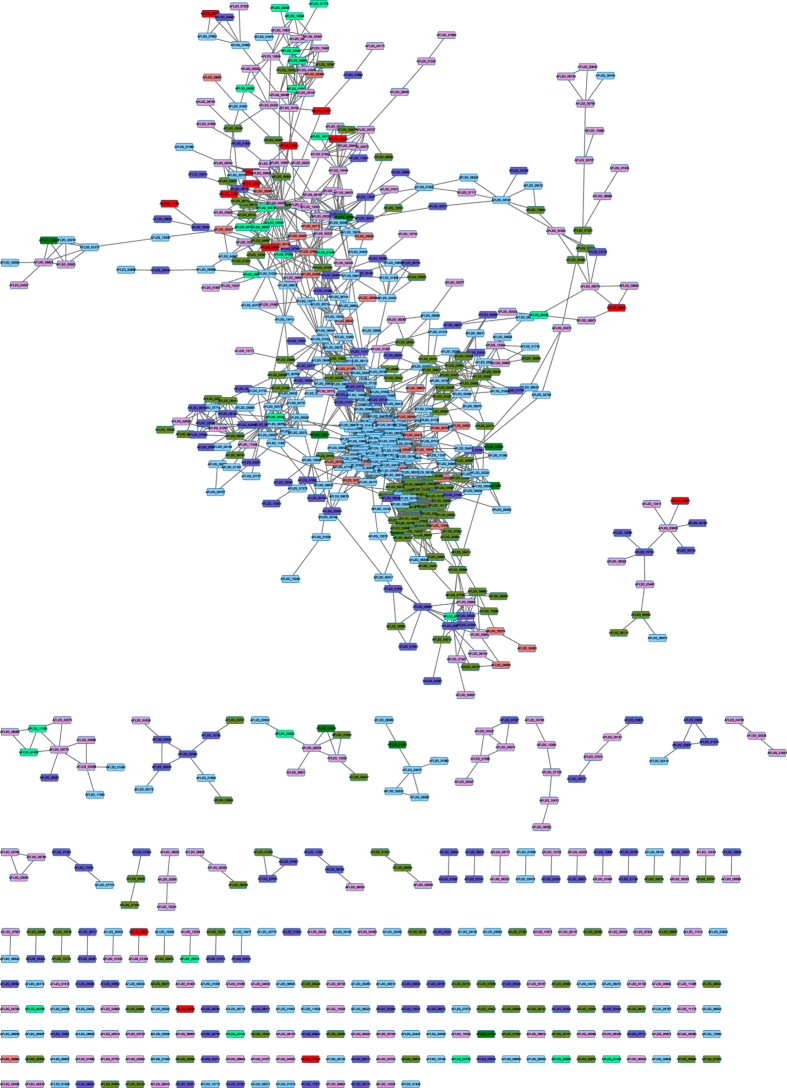
The protein-protein interaction network of *A. flavus* in response to tempmerature change. The PPI interactions with a combined score larger than 0.7 in the STRING database were extract to build the network. The gene with different regulatory pattern in protein/transcript level were marked as different color as follows: up/up, Red; down/down, Green; up/down, LightCoral; down/up, MediumSpringGreen; unchange/up, Plum; unchange/down, LightSkyBlue; up/unchange, OliveDrab; down/unchange, SlateBlue.

Most proteins exerted their biological functions by interacting with each other. To uncover functional aspects associated with these proteins, we constructed a PPI network based on data downloaded from the STRING database. Only protein pairs with a confidence score that was >0.7 were utilised to construct the PPI network; of these paris, 2,140 proteins were identified in our proteome data. By excluding the proteins without quantified or differential expression, our resulting PPI network contained 782 nodes and 3589 edges (Fig. 5).

According to the differential expression pattern on the transcript level and protein level at 28 °C compared to 37 °C, these nodes in the PPI network were divided into eight groups. We described four groups with differential expression of both the transcript and protein level. Group 1 contained 62 genes with up-regulated expression of both transcript and protein level at 28 °C. GO analysis revealed that group 1 genes were enriched in toxin biosynthesis and metabolic processes, as well as oxidation reduction. Group 2 contained 40 genes with down-regulated expression of both the transcript and protein level at 28 °C. Group 2 genes were enriched in “Choline metabolic process”, “Elastin metabolic process” and many processes involved in the immune response. Group 3 contained 48 genes with up-regulated expression of the protein level and a down-regulated transcript level at 28 °C. These genes were mainly enriched in the amino acid metabolic process. Group 4 contained 60 genes with up-regulated expression of the transcript level and a down-regulated protein level at 28 °C. These genes were mainly enriched in the proteolysis process and several subcategories of “carbohydrate metabolic process”, such as “glycoside biosynthetic process”.

### Correlation of the secondary metabolite clusters

Quantification of both mRNA and protein level changes allowed us to investigate the expression pattern of proteins and transcripts related to the secondary metabolite clusters. To evaluate the protein level changes of gene clusters related to secondary metabolite biosynthesis, 55 clusters were examined. Of these clusters, only 29 proteins showed differential protein level changes, which were mapped to eight clusters (cluster 10, 21, 23, 45, 47, 48, 54, and 55) (Supplementary Table S2). We also examined the correlation between changes in the transcript and protein level of genes of each cluster. Genes in cluster 54 were significantly correlated (*r* = 0.57, P = 3.9e-02). We also observed a positive correlation for cluster 1, 2, 4, and 5, although the rule of these clusters in *A. flavus* has not been explored.

### Aflatoxin biosynthesis-related proteins

**Figure 6:**
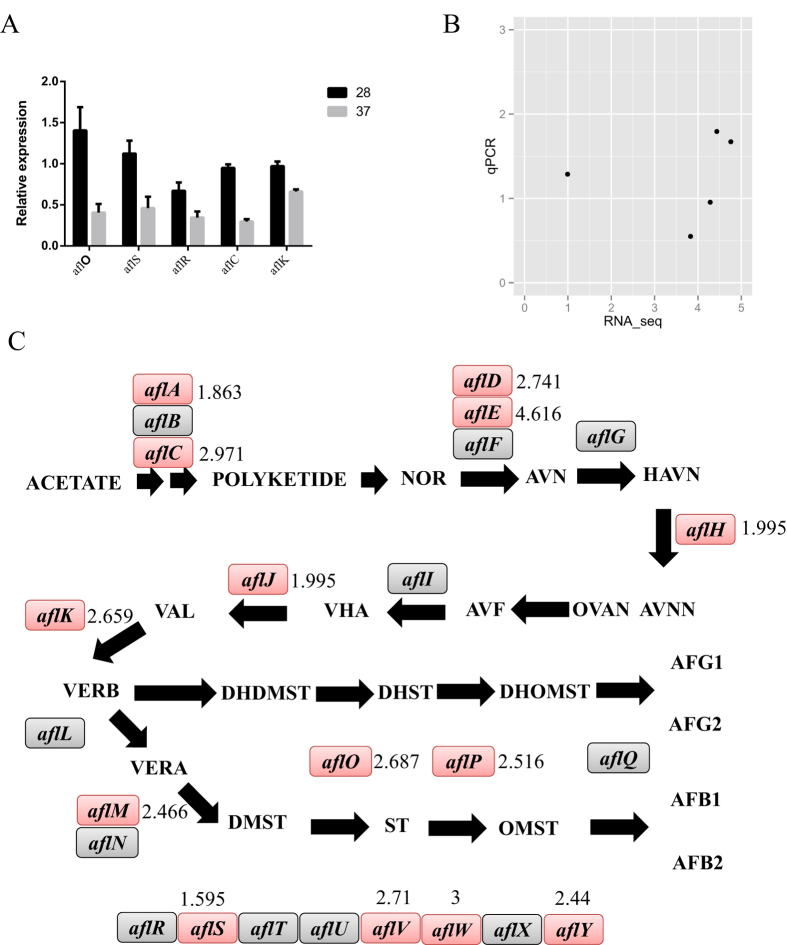
The regulation of aflatoxin biosynthesis related genes. (**A**) qPCR validation of the up-regulation of five aflatoxin biosynthesis genes (*aflC, aflK, aflO, aflS* and *aflR*) at 28 °C compared with 37 °C. (**B**) The correlation between the qPCR and RNA-seq data (in log2 format). (**C**) The quantification of fold changes in protein level of the aflatoxin biosynthesis genes.

To further support the observed changes in aflatoxin biosynthesis-related gene transcript levels, we chose three aflatoxin biosynthesis structure genes (*aflC, aflK, aflO*) and two aflatoxin biosynthesis regulatory genes (*aflS* and *aflR*) for additional analyses by quantitative real-time polymerase chain reaction (q-PCR). The results showed that all five transcripts were up-regulated at 28 °C (Fig. 6A), and there was agreement between the q-PCR and RNA-seq data(Fig. 6B). For example, the transcript level of *aflR* gene, a major regulatory gene of aflatoxin biosynthesis, was up-regulated by a fold change of 4.28 (RNA-seq data) and 1.93 (q-PCR) at 28 °C compared with 37 °C (Table 2).

Of 33 aflatoxin biosynthesis-related proteins, 12 proteins (aflE, aflW, aflC, aflD, aflO, aflP, aflK, aflM, aflY, aflJ, aflS, and aflH) were quantified by the iTRAQ method and showed a significantly higher protein level at 28 °C than at 37 °C (Fig. 6C; Table 2). The aflA and aflV proteins showed fold changes larger than 1.8, but these changes were not statistically significant (P > 0.05). The aflR protein was not detected in the proteomic profiles at different temperatures, which was similar to the observation in the *A. flavus* response to different water activities^11^. This suggested that the changes in *alfR* transcript expression change is a better marker of the transcript level than the protein level to investigate the activation of aflatoxin biosynthesis. Our data provided comprehensive and reliable transcriptome and proteome data of *A. flavus* at conducive and nonconducive temperatures.

## Discussion

Temperature is known to be a major environmental factor that influences aflatoxin production and has a great effect on the development of *A. flavus*. Although the transcriptome profiles of *A. flavus* in response to temperature changes (30 °C and 37 °C) have been reported^25^, our RNA-seq data provided more depth and coverage of gene expression in the *A. flavus* genome at different temperature (28 °C and 37 °C). We detected a greater number of DEGs at 28 °C and 37 °C, and half of the DEGs were identified using other samples^25^.

Our protein profile provides the most comprehensive information of proteins in *A. flavus* at 28 °C and 37 °C. Using the high-throughput method iTRAQ, we detected more than 30% of annotated proteins and quantified the relative expression level of 2,832 *A. flavus* proteins. Recently, our lab detected a similar number of expressed proteins at different water activities^11^. The proteome profiles of *A. flavus* grown in different conditions enriched the data resources for *A. flavus* and can be combined with several experiments that were conducted by using stable isotope labelling by amino acids in cell culture, two-dimensional electrophoresis and MS/MS^17^,^31^. The temperature difference did not result in a significant change in the relative abundance of more than half of *A. flavus* proteins, as reported previously^17^. In this study we identified more than 600 significant up/down-regulated proteins that were enriched on several KEGG pathways including the following: “Ribosome”, “Metabolic pathways”, “Biosynthesis of secondary metabolites”, three subsets of “Carbohydrate metabolism”, three subsets of “Amino acid metabolism”, “Linoleic acid metabolism” and “Methane metabolism”. The transcriptome analysis of *A. flavus* at 28 °C and 37 °C revealed that an elevated growth temperature altered amino acid metabolism. This was confirmed by our protein expression profiling and PPI network analysis; many proteins involved in translation and amino acid metabolism were highly up-regulated at 28 °C compared with 37 °C.

A low correlation between transcript level and protein concentration was detected in *A. flavus*, suggesting that the post-transcription modification process may play a critical role in the regulation of the final protein expression level. Many proteins (n = 274) identified were annotated with the following COG function categories: post-translational modification, protein turnover, and chaperones. There were many cases where protein expression changes were different from the transcript level changes. Smith *et al.* reported that many proteins whose concentration changed in response to temperature were encoded by corresponding RNA transcripts whose expression did not appear to change^15^. We also found that about 16% of genes encoding differentially expressed proteins had transcript accumulation and protein accumulation for the same gene that were in direct conflict with one another (Fig. 3C).

In this study, we found that 29 proteins located in secondary metabolite gene clusters (cluster 10, 21, 23, 45, 47, 48, 54 and 55) were differentially expressed at 28 °C and 37 °C. Cluster 48 is involved in the production of two related piperazines^32^. Cluster 54 plays a role in the production of aflatoxin^33^, and cluster 55 plays a role in the production of cyclopiazonic acid^34^.

For aflatoxin biosynthesis cluster 54, the backbone gene *aflR* encodes a DNA-binding, zinc-cluster protein that binds a palindromic sequence (TCGN5CGA) in the promoter region of aflatoxin pathway genes. The pathway-specific regulatory gene *aflR* is an absolute requirement for the activation of most aflatoxin pathway genes^35^. However, we could not detect aflR by our iTRAQ method, a similar result was reported by Georgianna *et al.* and Zhang *et al.*^11^,^17^. However, both the RNA-seq and q-PCR data confirmed that *aflR* was up-regulated in low temperature conditions. Therefore, we suggest that the changes in the *aflR* transcript level is a better marker for the activation of aflatoxin biosynthesis than the protein level.

## Conclusions

We compiled a comprehensive data set of protein and transcript expression changes that occur in *A. flavus* grown in conducive and nonconducive temperatures. We demonstrated that there was a low correlation between the proteome and transcriptome data, suggesting that post-transcriptional gene regulation influences different biological pathways and secondary metabolite gene clusters.

## Methods

### Strains and sample preparation

The *A. flavus* sample was prepared as described in our previous study^12^. Briefly, the standard cultivation of *A. flavus* strain NRRL 3357 was performed on yeast extract sucrose (YES) agar (20 g L^−1^ yeast extract, 150 g L^−1^ sucrose, and 15 g L^−1^ agar). Spores (10^6^) were inoculated onto the YES medium plate and incubated in the dark at 37 °C for 1.5 days (d) and 28 °C for 3 d to obtain the same amount of biomass. The aflatoxin production of *A. flavus* at 28 °C was 4.833 ± 1.041 μg · g^−1^, while that at 37 °C was 1.833 ± 0.577 μg · g^−1^.

### Protein preparation and iTRAQ labeling

*A. flavus* proteins were prepared according to our previous study^11^. Briefly, fungal samples were resuspended in lysis buffer supplemented with protease inhibitor solution and sonicated on ice. The expected proteins were extracted after centrifugation and precipitation. Each 100 μg of protein was digested in trypsin solution (1:10) and incubated at 37 °C for 12 h. The digested peptides were labelled using iTRAQ reagents according to the manufacturer’s instructions (Applied Biosystems, Foster City, CA, USA). The peptides from 37 °C and 28 °C were labelled with 114, 116, and 117 and 118, 119, and 121 iTRAQ reagents, respectively.

### Peptide separation and liquid chromatography–electrospray ionization–MS/MS analysis

To decrease the complexity of the labelled pepides, the mixture was separated by strong cation exchange chromatography using a Shimadzu HPLC system (LC-20AB; Shimadzu, Kyoto, Japan) as described previously in^36^. After reconstituting dried fractions with solvent A (5% acetonitrile [ACN] and 0.1% formic acid [FA]) to a concentration of 0.5 μg · μL^−1^, 10-μL samples were loaded on a Shimadzu LC-20AD nanoHPLC by the autosampler onto a 2-cm C18 trap column (inner diameter 200 μm). The peptides were eluted onto a resolving 10-cm analytical C18 column (inner diameter 75 μm) made in-house^37^. The liquid chromatography gradient consisted of 5% Solvent B (95% ACN and 0.1% FA) for 5 min, 5–35% Solvent B for 35 min, 60% Solvent B for 5 min, 80% Solvent B for 2 min, and 5% Solvent B for 10 min. Peptide-mixture MS data were acquired using a TripleTOF 5600 system (AB Sciex, Concord, Ontario, Canada) fitted with a Nanospray III source (AB Sciex) and a pulled quartz tip as the emitter (New Objectives, Woburn, MA). Data were acquired using an ion spray voltage of 2.5 kV, curtain gas of 30 PSI, nebulizer gas of 15 PSI, and an interface heater temperature of 150 °C. The MS was operated with a reversed-phase of greater than or equal to 30,000 full width at half maximum for time-of-flight MS scans. For information-dependent acquisition, survey scans were acquired in 250 ms, and as many as 30 product ion scans were collected if they exceeded a threshold of 120 counts per second (counts/s) and had a 2+ to 5+ charge state. The total cycle time was fixed to 3.3 s. The Q2 transmission window was 100 Da for 100%. Four time bins were summed for each scan at a pulse frequency value of 11 kHz by monitoring the 40-GHz multichannel time-to-digital converter detector with a four-anode channel detector ion. A sweeping collision energy setting of 35 ± 5 eV adjust rolling collision energy was applied to all precursor ions for collision-induced dissociation. Dynamic exclusion was set for half the peak width (18 s), and then the precursor was refreshed off the exclusion list.

### Proteomics data processing

Raw MS/MS data were converted into “.mgf” files using Proteinpilot software (AB Sciex). Mascot version 2.3.0 (Matrix Sciences, London, UK) was used to search against 12,604 annotated protein sequences of *A. flavus* that were downloaded from the Broad Institute Aspergillus Genomic Database (2013-05-16). The parameters were set as follows: peptide tolerance, 0.05 Da; fragment MS tolerance, 0.1 Da; fixed modification, methylthio (C); and variable modifications including oxidation (M), acetyl (N-term), pyro-glu (N-term Q), deamidation (N, Q), and iTRAQ 8-plex (N-term, K, Y). A maximum of one missed cleavage was allowed, and peptide charge states were set to +2 and +3. The search that was performed in Mascot was an automatic decoy database search. To identify false positives, raw spectra from the actual database were tested against a generated database of random sequences. Only peptides with scores significant at the 95% confidence level were considered to be reliable and were used for protein identification. For protein quantitation, a protein was required to contain at least two unique peptides. Protein quantitative ratios were weighted and normalized relative to the median ratio in Mascot. Only proteins with significant quantitative ratios between the two treatments (P < 0.05) and with fold changes >1.2 or <0.83 were considered to be differentially expressed.

### RNA sequencing

Total RNA was treated with DNase I to remove DNA, and magnetic beads with oligo (dT) were used to isolate mRNA. The mRNA was mixed with fragmentation buffer and fragmented into short fragments. Then the cDNA was synthesized using the mRNA fragments as templates. Short fragments were purified and resolved with EB buffer for end reparation and the addition of a adenine nucleotide. The short fragments were connected with adapters, and suitable fragments were selected for PCR amplification as templates. During the quality control steps, an Agilent 2100 Bioanalyzer and an ABI StepOnePlus Real-Time PCR System were used to quantify and qualify the sample library. Finally, the library was sequenced using the Illumina HiSeq 2000 sequencer (Beijing Genomics Institute, Shenzhen, Guangdong, China). After sequencing, raw image data were transformed by base calling into sequence data, which were called raw data or raw reads and were stored in Fastq format.

### Transcriptome data processing

After reads containing sequencing adapters and low-quality reads (quality value <20) were removed, the remaining reads were aligned to the *A. flavus* genome using the Tophat2 tool^24^. The expression value FPKM of each annotated transcript was calculated using the Cuffquant package of the Cufflinks program^24^.

### PPI network analysis

The PPI data of *A. flavus* were downloaded from the STRING database^38^. Each interaction has a combined score, which represents the reliability of the interaction between proteins. The PPI interactions with a combined score (0: lowest confidence; 1: highest confidence) larger than 0.7 were used for further network analysis. All differentially expressed proteins were mapped onto the PPI network and Cytoscape tool^39^ was used to visualise the network. GO term enrichment was determined by using the BiNGO plugin^40^ in Cytoscape.

### Quantitative reverse transcription-PCR analysis

Total RNA was isolated from mycelia harvested from 37 °C and 28 °C liquid stationary cultures using the EastepTM total RNA extraction kit (Promega, Shanghai, China) according to the manufacturer’s recommendations. DNA contamination was removed from the total RNA. One microgram of DNase-treated RNA was reverse transcribed using a reverse transcription kit (Thermo Scientific, Lithuania). The expression of *aflO, aflS, aflR, aflC* and *aflK* was analysed by q-PCR. The reactions were performed with Piko Real 96 (Thermo Scientific) using SYBR Green Jumpstart Taq Ready mix (Sigma). The relative expression was calculated using the 2^−ΔΔCT^ method as described by Schmittgen and Livak^41^. Tubulin gene expression was used as an internal reference.

## Additional Information

**How to cite this article**: Bai, Y. *et al.* Integrative analyses reveal transcriptome-proteome correlation in biological pathways and secondary metabolism clusters in *A. flavus* in response to temperature. *Sci. Rep.* **5**, 14582; doi: 10.1038/srep14582 (2015).

## Supplementary Material

Supplementary Figure S1

Supplementary Table S1

Supplementary Table S2

## Figures and Tables

**Table 1:** KEGG pathways enrichment analysis of differential expressed proteins.

Second-level KEGG pathway	Third-level KEGG Pathway	P value	Adjusted p value
Translation	Ribosome	3.04e-04	4.25e-03
Global and overview maps	Metabolic pathways	6.19e-04	4.33e-03
Carbohydrate metabolism	Glyoxylate and dicarboxylate metabolism	9.50e-04	4.43e-03
Carbohydrate metabolism	Fructose and mannose metabolism	1.57e-03	5.27e-03
Carbohydrate metabolism	Butanoate metabolism	1.88e-03	5.27e-03
Amino acid metabolism	Histidine metabolism	5.12e-03	1.08e-02
Lipid metabolism	Linoleic acid metabolism	5.38e-03	1.08e-02
Energy metabolism	Methane metabolism	8.74e-03	1.53e-02
Amino acid metabolism	Valine, leucine and isoleucine degradation	1.08e-02	1.64e-02
Global and overview maps	Biosynthesis of secondary metabolites	1.17e-02	1.64e-02
Metabolism of cofactors and vitamins	Biotin metabolism	1.74e-02	2.06e-02
Amino acid metabolism	Glycine, serine and threonine metabolism	1.76e-02	2.06e-02
Metabolism of cofactors and vitamins	Vitamin B6 metabolism	2.04e-02	2.19e-02

**Table 2:** The expression changes of genes on aflatoxin biosynthesis cluster.

NCBI ID	Protein changes (28/37)	Significant	Transcripts changes log2(28/37)
aflE	4.616	*	4.183329
aflW	3	*	4.019371
aflC	2.971	*	4.758722
aflD	2.741	*	5.349907
aflV	2.71		3.573936
aflO	2.687	*	4.435909
aflK	2.659	*	3.828964
aflP	2.516	*	4.770821
aflM	2.466	*	4.139784
aflY	2.44	*	3.512412
aflJ	1.995	*	4.468144
aflS	1.595	*	0.992644
aflA	1.863		3.282081
aflH	1.995	*	4.468144
aflR	NA	NA	4.28307

NA: The protein was not detected by iTRAQ.
