# Supplementary material for: Integrative analyses reveal transcriptome-proteome correlation in biological pathways and secondary metabolism clusters in A. flavus in response to temperature
Source: Sci Rep. 2015 Sep 29;5:14582. doi: 10.1038/srep14582 (PMC4586720; doi:10.1038/srep14582)
Supplement: Supplementary Figure S1 [file srep14582-s1.doc]

### Integrative analyses reveal transcriptome-proteome correlation in biological pathways and secondary metabolism clusters in *A. flavus* in response to temperature

Youhuang Bai^#^, Sen Wang^#^, Hong Zhong^#^,Qi Yang, Feng Zhang, Zhenhong Zhuang, Jun Yuan, Xinyi Nie, Shihua Wang^*^

**SUPPLEMENTAL DATA**

**Supplementary Figure S1.** Replicate analyses of peptide quantitation by iTRAQ experiments. More than 90% coverage of our identified proteins expression values fell within 50% expression variation.

**Supplementary Table S**1**.** The detail information of proteins identified by the iTRAQ in A. flavus grown at 28 ^o^C and 37^o^C.

**Supplementary Table S2.** The detail information of differentially expressed proteins with transcript level expression in *A. flavus.*

**Supplementary Figure S1.** We categorized each iTRAQ result into groups of variation ranging from 10% to 100%, and found that more than 90% coverage of our identified proteins expression values fall within 50% expression variation
